# CEUS Helps to Rerate Small Breast Tumors of BI-RADS Category 3 and Category 4

**DOI:** 10.1155/2014/572532

**Published:** 2014-05-25

**Authors:** Jian-xing Zhang, Li-shan Cai, Ling Chen, Jiu-long Dai, Guang-hui Song

**Affiliations:** ^1^Departments of Ultrasound, Second Affiliated Hospital, Guangzhou University of Traditional Chinese Medicine, Guangzhou 510120, China; ^2^Departments of Ultrasound, First Affiliated Hospital, Guangzhou University of Traditional Chinese Medicine, Guangzhou 510405, China

## Abstract

*Purpose*. The primary aim of this study was to explore if classification, whether using the BI-RADS categories based on CEUS or conventional ultrasound, was conducive to the identification of benign and malignant category 3 or 4 small breast lesions. *Material and Methods*. We evaluated 30 malignant and 77 benign small breast lesions using CEUS. The range of enhancement, type of enhancement strength, intensity of enhancement, and enhancement patterns were independent factors included to assess the BI-RADS categories. *Results*. Of the nonenhanced breast lesions, 97.8% (44/45) were malignant, while, of the hyperplasic nodules, 96.8% (30/31) showed no enhancement in our study. Category changes of the lesions were made according to the features determined using CEUS. The results showed that these features could improve diagnostic sensitivity (from 70.0 to 80.0, 80.0, 90.0, and 90.0%), reduce the negative likelihood ratio (from 0.33 to 0.22, 0.25, 0.11, and 0.12), and improve the NPV (from 88.8 to 92.2, 91.2, 96.2, and 95.5%). However, this was not conducive to improve diagnostic specificity or the PPV. *Conclusion*. The vast majority of nonenhanced small breast lesions were malignant and most of the hyperplasic nodules showed no contrast enhancement. As a reference, CEUS was helpful in identifying BI-RADS category 3 or 4 small breast lesions.

## 1. Introduction

The American College of Radiology Breast Imaging Reporting and Data System (ACR BI-RADS) proposed ultrasound breast disease standardized diagnostic criteria in 2003 [1]. However, its low specificity has been troubling to clinicians [2, 3]. In the ACR-BI-RADS-US criteria, category 3 (less than 2 percent risk of malignancy) or category 4 (probability of cancer, ranging from 3 to 94 percent) lesions are considered different degrees of malignant breast lesions. This is especially true for hyperplastic nodules in category 3, which are considered to be uncertain ones. Such lesions do not have obvious characteristics of benign lesions, but they are still considered subjectively as category 3 lesions. There are 1-2 nonbenign characteristics of category 4 lesions, but the ACR does not provide any detailed guidance. This leads to poor interobserver consistency in classification. As one study shows [4], in some of the diagnostic category 4 subclasses, consistency is poor, especially for category 4c. This poor reproducibility reflects the highly subjective nature of this classification.

Contrast-enhanced ultrasound (CEUS) of the breast is less commonly performed than abdominal CEUS [5, 6]. In previous studies, ultrasound contrast medium was used to help differentiate between benign and malignant breast disease. However, use of this technique did not always give uniform results [7–9]. Early breast cancer detection, diagnosis, and treatment have been shown to be the key to improving the cure rate and reducing mortality [10]. On clinical exam, the current discovery rate of detection of early breast cancer in the United States is 25%, so accurate ultrasound diagnosis of early breast cancer is imperative to improve early detection; however, diagnosis of early breast cancer using ultrasound is difficult [10]. There is a higher rate of misdiagnosis and missed diagnosis of small size, nonpalpable, and atypical ultrasonographic early stage breast cancer, especially when the maximum diameter of the lesion is ≤10 mm (cancer clinical stage belongs to T1a and T1b) [11]. The ability to diagnose early stage breast cancer when lesions are ≤10 mm would have important clinical value by improving cure rates.

There have been no published studies done on breast cancer lesions in the diameter range of 1–10 mm. On the basis of previous studies, we have summarized the characteristics of CEUS used in the detection of benign and malignant breast lesions. We have also explored the clinical value of ultrasound technology in diagnosing breast cancer at the early and malignant stages when nodules are classified as T1a or T1b and belong to BI-RADS-US category 3 or 4. At the same time, this study also explored whether the corrected BI-RADS category was appropriate in identifying breast lesions at the early and malignant stages based on CEUS.

## 2. Materials and Methods

### 2.1. Patients

Approval from the Regional Ethics Committee was obtained for this study. Written informed consent was obtained from all patients participating in this study. A total of 107 patients (each with one lesion) were evaluated from June 2011 to April 2013. All patients underwent conventional preoperative ultrasonography (US) and CEUS. The results showed that all nodules belonged to category 3 or 4 using US, and the size of the lesions ranged from 1 to 10 mm.

None of the lesions were biopsied before contrast studies were performed. The inclusion criterion was the presence of lesions on US. The exclusion criterion was contraindication to contrast agent administration. The contrast agent was not given if patients had heart disease, pulmonary or respiratory disease, or hypertension, were allergic to the contrast agent, or were pregnant or breast-feeding.

All patients underwent surgery and had a final pathologic diagnosis. Malignant lesions were found in 30 patients (age range from 31 to 71 years old and mean age was 49 years old), and 77 patients had benign lesions (age range from 25 to 64 years old and mean age was 44 years old) according to the histopathology.

### 2.2. Conventional and Contrast-Enhanced Imaging Protocol

GE LOGIQ E9 and LOGIQ 9 systems (GE Healthcare, USA) with a linear array transducer (LOGIQ 9 M12L or LOGIQ E9 ML6–15) were used for conventional US. A 4–9 MHz linear transducer was used for CEUS. The contrast agent was SonoVue (Bracco SpA, Milan, Italy), a lyophilized powder of phospholipid-stabilized microbubbles containing sulfur hexafluoride gas with a mean diameter of 2.5 *μ*m. The solution was reconstituted by the addition of 5 mL of sterile saline. B-mode pulse inversion harmonic imaging was used in CEUS. Settings were as follows: mechanical index was 0.12-0.13, image depth was 3 or 4 cm, the single focus was at the bottom of the image, and the probe was stabilized manually and no pressure was exerted.

One ultrasound physician (JX Zhang with 15 years of experience in breast US) performed all US and CEUS examinations. On the US, the maximum imaging plane of the mass, which included the mass and its surrounding normal tissue (if it was possible), was selected for CEUS. After a manual bolus injection of 2.4 mL of SonoVue via a 20-gauge cannula placed in the antecubital vein, the selected plane remained unchanging during the examination and real-time imaging was recorded for up to 3 min. All static and dynamic images were stored in the ultrasound systems, and then single frames in JPEG format and movie files in digital imaging communications in medicine (DICOM) format were exported to a personal computer.

### 2.3. Pathological Method

A Nikon 80i upright fluorescence microscope, a Shandon Pathcentre tissue processor, a Leica 2145 rotary microtome, and a Leica ST5030 multistainer workstation were used in the pathological examination.

### 2.4. Image Analysis

All dynamic contrast images and conventional images were stored on the hard drive of the machine. We analyzed the CEUS-detected changes in malignant and benign lesions including contrast-enhanced range, enhanced type, intensity of enhancement, and the pattern of enhancement.

Two ultrasound physicians evaluated all ultrasonographic images without knowledge of patient clinical data, and disagreements were resolved by a third-party appraisal [6, 12].

**Figure 1:**
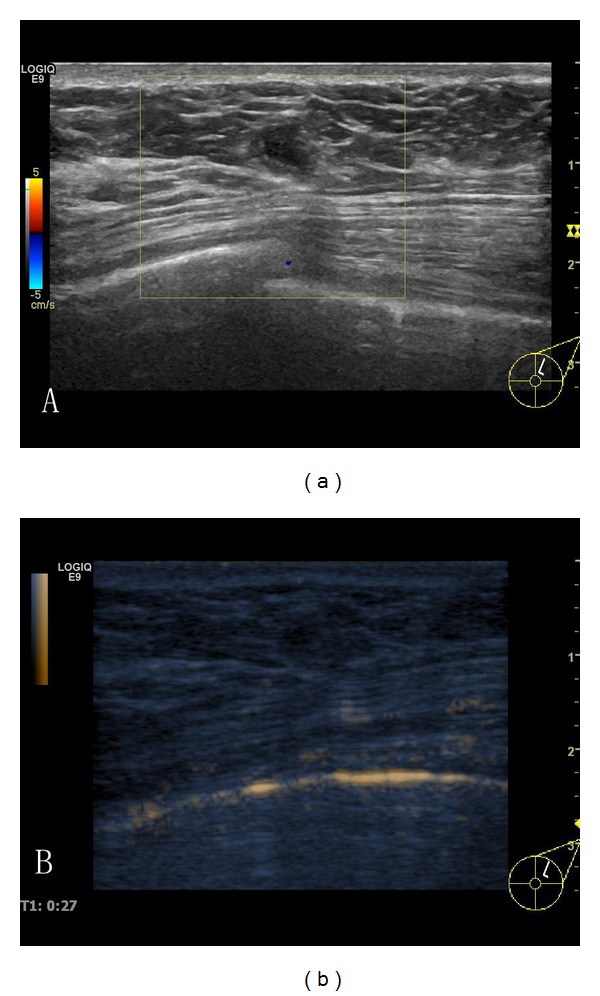
A sonogram of a right breast from a 42-year-old woman is shown. (a) There was a solid hypoechoic nodule of 5 × 4 mm at 12-1 o'clock in the right breast, with a circumscribed margin and defined, irregular shape, with a heterogeneous internal echo and nodular posterior echo enhancement. CDFI (color Doppler flow imaging): there were no color flow signals in and surrounding the nodule. Ultrasound diagnosis: BI-RADS 4a. CEUS (b): there was no significant enhancement in and surrounding the whole of the nodule. CEUS diagnosis: BI-RADS 3. Pathology: hyperplastic disease.

**Figure 2:**
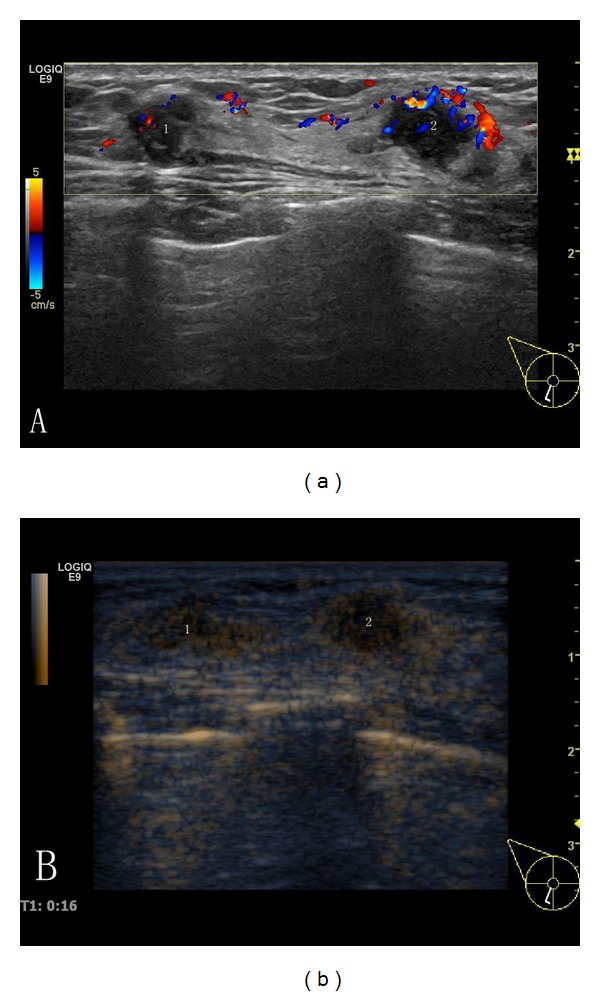
A sonogram of a right breast from a 57-year-old woman is shown. (a) There were two solid hypoechoic lesions at 7 o'clock in the right breast (lesion 1: 6 × 5 mm and lesion 2: 9 × 7 mm), with a circumscribed margin and defined, irregular shape, with a heterogeneous internal echo and nodular posterior echo enhancement. CDFI (color Doppler flow imaging): rich color flow signals were clearly visible in the nodule and its surrounding. Ultrasound diagnosis: BI-RADS 4c. CEUS (b): in the early arterial phase, nodules within its surrounding with significantly heterogeneous concentric enhancement can be seen. The range of strengthening was greater than the range that was in US. CEUS diagnosis: BI-RADS 5. Pathology: invasive ductal breast carcinoma.

When results of the echo strength range of the lesion size was less than 17 mm, it was regarded that the range, which was 3 mm larger than the original one, would be considered as the enlarged strength range [13] (Figure 1). When the range was less than that from US or with no enhancement, it was regarded as low-enhancement (Figure 2). In addition to these types of lesions, there were those that were regarded as having equal-enhancement.

Contrast-enhanced images were classified into three categories according to the distribution of enhanced types of the mass: (1) no enhancement, with lack of enhancement after contrast agent injection; (2) homogeneous enhancement, in which diffuse and homogeneous enhancement was apparent across the whole mass; and (3) inhomogeneous enhancement, in which enhancing areas were only in or more confined to the mass periphery; or there was part of the mass that showed homogeneous enhancement; or the mass was heterogeneously enhanced [6, 14, 15].

According to the pattern of enhancement using CEUS, masses were classified into four categories: (1) no enhancement; (2) centripetal enhancement, in which they were enhanced from edge to center; (3) centrifugal enhancement, in which they were enhanced from center to edge; and (4) atypical enhancement, in which they were not typically enhanced [6, 12, 13].

According to the intensity of enhancement using CEUS, contrast-enhanced images were classified into four categories: (1) no enhancement; (2) high-enhancement, in which the lesions were more highly enhanced than that of normal tissue; (3) low-enhancement, in which the lesions were less enhanced than that of normal tissue; and (4) equal-enhancement, in which lesions and normal tissues were equally enhanced [14, 16, 17].

In the literature [6, 14–18], high-enhancement of the lesion, strength and heterogeneous enhancement, and peripheral radial enhancement were considered as standards of breast tumor enhancement. Lesions with the signs mentioned above were regarded as a higher classification of a malignant lesion. Lesions with low malignant-potential changes were converted into the corresponding category of low malignant-potential, which included no enhancement, homogeneous enhancement, centripetal enhancement, and low-enhancement. Others were retained in their original categories. Those have also been described in “*The clinical application guideline of CEUS*” which was proposed by the Chinese Medical Association Sonographer Branch.

### 2.5. Statistical Analysis

Statistical analysis was conducted using the SPSS statistical package, version 19.0 for Windows (SPSS Institute, Cary, NC, USA). According to the gold standard of pathological diagnosis, we comparatively analyzed the categories used in US with the corrected categories used in CEUS. The results of our study were evaluated on the basis of a statistical compilation and diagnostic test for statistically assessing the discriminatory power of US and CEUS.

## 3. Results

All 107 lesions were solid-appearing hypoechoic lesions. Thirty lesions were malignant and 77 lesions were benign on histopathology. Malignant lesions ranged in size from 4 to 10 mm (8.73 mm standard deviation). Benign lesions ranged in size from 3 to 10 mm (7.52 mm standard deviation). Two malignant lesions and 10 benign lesions ranged in size from 3 to 5 mm. There was no significant difference in tumor size between malignant and benign lesions.

Based on US, there were 31 cases of category 3, 49 cases of category 4a, six cases of category 4b, and 21 cases of category 4c in this study. Forty-five lesions showed no obvious contrast enhancement. In those with no enhancement lesions, there were 23 cases of category 3, 21 cases of category 4a,and one case of category 4b.

### 3.1. Histopathological Findings

There were nineteen cases of invasive ductal carcinoma (IDC), two cases of tubular carcinoma, one case of invasive lobular carcinoma, four cases of mucinous carcinoma, two cases of ductal carcinoma* in situ* (DICS), and two cases of papillary carcinoma with minimal invasion. In the 77 cases of benign lesions, there were 19 fibroadenomas, nine intraductal papillomas, 47 hyperplastic breast lesions (one case of a hyperplastic nodule around the catheter with inflammation, 10 breast adenosis tumors, 31 hyperplastic nodules, three ductal papillary hyperplasias, and one moderate-to-severe dysplasia), one infected cyst, one postoperative scar, and one case of lipoma.

Among these 45 lesions without enhancement, 44 were benign (30 hyperplastic nodules, three breast adenosis tumors, two papillomas, five fibroadenomas, one postoperative scar, two ductal papillary hyperplasias, and one moderate-to-severe dysplasia) and one malignant case (mucinous carcinoma).

### 3.2. BI-RADS Regulate Category on CEUS

We modified the category of BI-RADS according to the contrast enhancement features using CEUS, which included the range of enhancement (Table 1), type of enhancement (Table 2), intensity of enhancement (Table 3), and pattern of contrast enhancement (Table 4).

Fifty-seven benign lesions with no enhancement or range of enhancement less than that of US modified to the low malignant-potential category as shown in Table 1 and two benign lesions and six malignant lesions with enlarged enhancement modified to the high malignant-potential category; the others were kept in their original category.

Forty-five benign lesions with no enhancement modified to the low malignant-potential category as shown in Table 2, and 17 benign lesions and 16 malignant lesions with inhomogeneous enhancement modified to the high malignant-potential category; the others were kept in their original category.

Fifty-three benign lesions with no enhancement or homogeneous enhancement modified to the low malignant-potential category as shown in Table 3, and 13 benign lesions and 24 malignant lesions with inhomogeneous enhancement modified to the high malignant-potential category; the others were kept in their original category.

Forty-four benign lesions and seven malignant lesions with no enhancement or centrifugal enhancement modified to the low malignant-potential category as shown in Table 4, and 14 benign and 18 malignant lesions with centripetal enhancement modified to the high malignant-potential category; the others were kept in their original category.

### 3.3. Different Diagnosis between US and CEUS

Comparisons of the pathological diagnosis on the basis of the determined category 4b in benign and malignant breast tumor were shown in Table 5. The judgment study of benign and malignant breast lesions was shown in Table 6.

## 4. Discussion

Small lesions of early breast cancer are nonpalpable, especially if the size of lesion is between 1 and 10 mm (stages T1a and T1b). These lesions have no typical ultrasonographic features and there is a high rate of misdiagnosis and missed diagnosis [19]. Meanwhile, tumor sizes ≤10 mm are also difficult to diagnose and are mainly category 3 or 4 cases. If these tumors were malignant, they were still in DICS Stage or in the early stages of invasion. Early detection has a much more positive impact on the treatment, prognosis, and survival rate of breast cancer patients [20]. Early diagnosis of the disease also determines the treatment options described in the National Comprehensive Cancer Network (NCCN) guidelines. However, there are numerous differences between treatments for T1a, T1b, and other size tumors [11]. This study not only helps to clearly identify the type of lesion but also can lead to a reduction of unnecessary biopsies or reviews. At the same time, it also reduces patient suffering and improves the effectiveness and use of medical resources.

Malignant tumors of the breast have been shown to be readily visualized owing to their contrast medium staining [21–25]. This property has been exploited in contrast medium-enhanced magnetic resonance tomography, which is known, however, for its high sensitivity and low specificity in the identification of carcinomas [26, 27]. During the past decade, the introduction of sonographic microbubble contrast agents has offered an option to improve the ability of detecting the blood flow signal [28–31].

Nonenhanced ultrasound is considered to have a high specificity but a low sensitivity for breast cancer. In our study, 45 lesions showed no obvious contrast enhancement over the entire observed area. Of those cases, most of them were hyperplasic nodules and 96.8% (30/31) of hyperplasic nodules showed no enhancement, consistent with previous studies [32]. It was helpful to change the category of BI-RADS used in the diagnosis of benign and malignant lesions that was based on nonenhanced lesions using CEUS. But it must be noted that one case with no enhancement was a mucinous adenocarcinoma, which has also been reported in the literature [14].

It should be clarified that most investigators have focused their studies on the discrimination between benign and malignant masses [20, 23, 26, 30, 31, 33, 34]. We changed the category of BI-RADS that referred to enhancement features using CEUS [6, 12–18]. In this study, 25 lesions were changed from category 3 to category 2 and 32 lesions from category 4a to category 3, according to the range of enhancement determined using CEUS. All of these lesions were benign. This led to a reduction of unnecessary biopsies or reviews. It also improved the sensitivity (from 70 to 80%), accuracy (from 86.0 to 88.8), PPV (from 77.8 to 80.0%), and NPV (from 88.8 to 92.2%) and reduced the negative likelihood ratio from 0.33 to 0.22. More malignant breast lesions enlarged the range of enhancement using CEUS than using US, while more benign lesions did not [6]. It was easy to identify the range of lesions using CEUS for small lesions. This could increase accuracy of judgments in line. However, the range of enhancement was not enlarged in three malignant lesions, two mucinous carcinomas and one DCIS.

We must point out that most lesions in the modified category were based on lesions without enhancement. Changes also happened in the modified category according to the strength type of enhancement; 20 lesions were changed from category 3 to category 2, and 20 lesions from category 4a to category 3. This improved the sensitivity and reduced the negative likelihood ratio (from 0.33 to 0.25) but did not improve specificity. In these groups, heterogeneous enhancement did not appear in all malignant cases, contrary to what has been described in the literature, whereby an unbalanced spatial distribution of tumor blood vessels could lead to heterogeneous enhancement [19]. It is possible that the reason we did not see heterogeneous enhancement was that the lesions, which were <10 mm, were too small. Thus, there was no significant difference in enhancement of the strength type between benign and malignant lesions, which affected the accuracy of diagnosis using the BI-RADS categories. Another issue was that subjectivity played a large role in determining whether there was heterogeneous enhancement or not in small lesions.

Tumor angiogenesis is the development of a new vascular network essential for tumor growth and infiltration [35, 36] and is the basis for CEUS. This is closely related to the microvessel density (MVD) and the strength of enhancement of the tumor using CEUS, as described in the literature [37–39]. In our study, we altered the categories of the BI-RADS using CEUS. Twenty-five lesions were changed from category 3 to category 2, and 32 lesions from category 4a to category 3. Furthermore, sensitivity (from 70 to 90%), accuracy (from 86 to 88%), and NPV (from 88.8% to 96.2%) all increased, and the negative likelihood ratio decreased (from 0.33 to 0.11); however, the PPV was reduced from 8.98 to 7.12. This study shows that malignant lesions also have a richer microvascular than that of benign lesions, even in early breast cancer [36]. Therefore, the strength of enhancement was more obvious using CEUS. There were some benign inflammatory nodule merger cases. Inflammatory responses could also be accompanied by local vascular dilation, which could increase. Two papilloma cases were also high-enhancement ones. Those could interfere with the correction based on categories.

Twenty-three lesions were changed from category 3 to category 2, and 17 lesions from category 4a to category 3, according to their enhancement patterns. This also resulted in an improvement in sensitivity (from 70 to 90%) and a reduction in the rate of negative likelihood ratio (from 0.33 to 0.12); however, specificity and accuracy were worse than with the BI-RADS-US. The presence of inflammatory nodules was one of the causes of misdiagnosis, as was the size of lesions, because it was difficult to identify centrifugal or centripetal enhancement in small lesions.

## 5. Conclusion

CEUS in breast lesion characterization is a new type of examination that is different from US. It can provide more details on the microvasculature and hemodynamics than US. We explored the value of the categories and the correction of the BI-RADS classification determined using CEUS or US. This was conducive to the identification of benign and malignant breast lesions. Of the no enhancement lesions, 97.8% (44/45) were malignant breast lesions and 96.8% (30/31) of hyperplasic nodules showed no enhancement in our study. Those cases played an important role in the decision to implement the category changes in this study.

The results showed that these features determined using CEUS could improve diagnostic sensitivity, reduce the negative likelihood ratio, and improve the NPV; however, this was not beneficial in improving the specificity of diagnosis and PPV. It can increase the negative likelihood ratio, in which category changes were made according to the strength and range of enhancement. Many benign lesions were changed from category 3 to 2 or from category 4a to 3, according to the features determined using CEUS. These were helpful in the identification of early breast cancer and can be used as a reference.

## Figures and Tables

**Table 1:** Changed category according to the range of enhancement in CEUS.

BI-RADS category	Changed BI-RADS category	Pathology	
		Benign (*n* = 77)	Malignant (*n* = 30)
3 (*n* = 31)	2 (*n* = 25)	25	0
	3 (*n* = 5)	5	0
	4a (*n* = 1)	1	0

4a (*n* = 49)	3 (*n* = 32)	32	0
	4a (*n* = 14)	8	6
	4b (*n* = 3)	0	3

4b (*n* = 6)	4b (*n* = 5)	3	2
	4c (*n* = 1)	1	0

4c (*n* = 21)	4c (*n* = 18)	2	16
	5 (*n* = 3)	0	3

**Table 2:** Changed category according to the type of enhancement in CEUS.

BI-RADS category	Changed BI-RADS category	Pathology	
		Benign (*n* = 77)	Malignant (*n* = 30)
3 (*n* = 31)	2 (*n* = 23)	23	0
	3 (*n* = 6)	6	0
	4a (*n* = 2)	2	0

4a (*n* = 49)	3 (*n* = 20)	20	0
	4a (*n* = 15)	9	6
	4b (*n* = 14)	11	3

4b (*n* = 6)	4a (*n* = 2)	2	0
	4c (*n* = 4)	2	2

4c (*n* = 21)	4c (*n* = 8)	0	8
	5 (*n* = 13)	2	11

**Table 3:** Changed category according to the intensity of enhancement in CEUS.

BI-RADS category	Changed BI-RADS category	Pathology	
		Benign (*n* = 77)	Malignant (*n* = 30)
3 (*n* = 31)	2 (*n* = 25)	25	0
	3 (*n* = 1)	1	0
	4a (*n* = 5)	5	0

4a (*n* = 49)	3 (*n* = 23)	23	0
	4a (*n* = 11)	9	2
	4b (*n* = 15)	8	7

4b (*n* = 6)	4a (*n* = 4)	3	1
	4b (*n* = 1)	1	0
	4c (*n* = 1)	0	1

4c (*n* = 21)	4b (*n* = 2)	2	0
	4c (*n* = 3)	0	3
	5 (*n* = 16)	0	16

**Table 4:** Changed category according to the pattern of contrast enhancement in CEUS.

BI-RADS category	Changed BI-RADS category	Pathology	
		Benign (*n* = 77)	Malignant (*n* = 30)
3 (*n* = 31)	2 (*n* = 23)	23	0
	3 (*n* = 6)	6	0
	4a (*n* = 2)	2	0

4a (*n* = 49)	3 (*n* = 19)	17	2
	4a (*n* = 14)	13	1
	4b (*n* = 16)	10	6

4b (*n* = 6)	4a (*n* = 3)	3	0
	4b (*n* = 1)	0	1
	4c (*n* = 2)	1	1

4c (*n* = 21)	4b (*n* = 6)	1	5
	4c (*n* = 3)	0	3
	5 (*n* = 12)	1	11

**Table 5:** Ultrasound diagnosis in US and CEUS.

	US	Range of enhancement	Type of enhancement	Intensity of enhancement	Enhancement pattern
A (true- positive)	21	24	24	27	27
B (false- positive)	6	6	15	11	13
C (false- negative)	9	6	6	3	3
D (true-negative)	71	71	62	66	64

**Table 6:** Ultrasound diagnostic study of BI-RADS classification in US and CEUS.

	Sensitivity %	Specificity %	Positive likelihood ratio	Negative likelihood ratio	Youden index %	Accuracy %	PPV %	NPV %
BI-RADS category	70.0	92.1	8.98	0.33	0.62	86.0	77.8	88.8
Range of enhancement	80.0	92.1	10.27	0.22	0.72	88.8	80.0	92.2
Type of enhancement	80.0	80.5	4.11	0.25	0.61	80.4	61.5	91.2
Strengthening of enhancement	90.0	87.4	7.12	0.11	0.77	88.0	71.1	96.2
Enhancement pattern	90.0	83.1	5.33	0.12	0.73	85.1	67.5	95.5

PPV: positive predictive value and NPV: negative predictive value.
